# Patients’ online access to psychiatric records: Providers’ experiences

**DOI:** 10.1192/j.eurpsy.2022.1462

**Published:** 2022-09-01

**Authors:** A. Fagerlund, P. Zanaboni, O. Lintvedt, E. Kristiansen, R. Wynn

**Affiliations:** 1 University Hospital of North Norway, Norwegian Centre For Ehealth Research, Tromsø, Norway; 2 UiT The Arctic University of Norway, Department Of Clinical Medicine, Tromsø, Norway

**Keywords:** digital health, e-health, Electronic Health Records

## Abstract

**Introduction:**

Patients attending psychiatric specialist health services in Northern Norway have since 2015 had the opportunity to access their medical records online. Prior to implementation, there were some concerns in the professional field that patient accessible electronic health records might introduce some challenges.

**Objectives:**

In this study, we asked psychiatric providers in outpatient psychiatric care about the impact of patients’ online access to documentation practices and whether they felt the access impacted the provider-patient relationship. We also examined whether the providers sought to deny patients’ access to any information.

**Methods:**

16 qualitative in-depth interviews were performed with mental health providers working in the specialist services in North Norway. Participants had different professional backgrounds, and included doctors, nurses, psychologists and others. The interviews were audio recorded and transcribed verbatim. The data were qualitatively analyzed by means of the framework method.

**Results:**

The providers varied in their encouragement of patients’ online access, but few expressed concerns. There had been little specific training on how to optimize the writing of notes to accomodate patients’ online acces, but several pointed out that there had been an increased focus on the importance of adapting the notes to promote understanding. Increased transparency was in general seen positively, but the service might not fit all patient categories. Very few patients were denied access. In most cases, the service could improve the patients’ understanding of the treatment and the provider-patient relationship.

**Conclusions:**

While some voiced caveats, patients’ online access was in general seen as beneficial to the treatment and the provider-patient relationship.

**Disclosure:**

No significant relationships.

